# FHOD1, a Formin Upregulated in Epithelial-Mesenchymal Transition, Participates in Cancer Cell Migration and Invasion

**DOI:** 10.1371/journal.pone.0074923

**Published:** 2013-09-26

**Authors:** Maria Gardberg, Katja Kaipio, Laura Lehtinen, Piia Mikkonen, Vanina D. Heuser, Kati Talvinen, Kristiina Iljin, Caroline Kampf, Mathias Uhlen, Reidar Grénman, Mari Koivisto, Olli Carpén

**Affiliations:** 1 Department of Pathology, University of Turku and Turku University Hospital, Turku, Finland; 2 Medical Biotechnology, VTT Technical Research Centre of Finland, and Turku Centre for Biotechnology, University of Turku, Turku, Finland; 3 Department of Immunology, Genetics and Pathology, Science for Life Laboratory, Uppsala University, Uppsala, Sweden; 4 Science for Life Laboratory and Albanova University Center Royal Institute of Technology, Stockholm, Sweden; 5 Department of Otorhinolaryngology – Head and Neck Surgery and Department of Medical Biochemistry and Genetics, Turku University Hospital and University of Turku, Turku, Finland; 6 Department of Biostatistics, University of Turku, Turku, Finland; Karolinska Institutet, Sweden

## Abstract

Cancer cells can obtain their ability to invade and metastasise by undergoing epithelial-to-mesenchymal transition (EMT). Exploiting this mechanism of cellular plasticity, malignant cells can remodel their actin cytoskeleton and down-regulate proteins needed for cell-cell contacts. The mechanisms of cytoskeletal reorganisation resulting in mesenchymal morphology and increased invasive potential are poorly understood. Actin nucleating formins have been implicated as key players in EMT. Here, we analysed which formins are altered in squamous cell carcinoma related EMT. FHOD1, a poorly studied formin, appeared to be markedly upregulated upon EMT. In human tissues FHOD1 was primarily expressed in mesenchymal cells, with little expression in epithelia. However, specimens from oral squamous cell cancers demonstrated consistent FHOD1 upregulation in mesenchymally transformed cells at the invasive edge. This upregulation was confirmed in an oral squamous carcinoma model, where FHOD1 expression was markedly increased upon EMT in a PI3K signalling dependent manner. In the EMT cells FHOD1 contributed to the spindle-shaped morphology and mesenchymal F-actin organization. Furthermore, functional assays demonstrated that FHOD1 contributes to cell migration and invasion. Finally, FHOD1 depletion reduced the ability of EMT cancer cells to form invadopodia and to degrade extracellular matrix. Our results indicate that FHOD1 participates in cytoskeletal changes in EMT. In addition, we show that FHOD1 upregulation occurs during cancer cell EMT *in vivo*, which indicates that FHOD1 may contribute to tumour progression.

## Introduction

The actin cytoskeleton is a highly plastic structure with ability to rapidly remodel upon many extra- and intracellular cues. In epithelial-to-mesenchymal transition (EMT), epithelial cells dissociate their cell-cell junctions, and remodel their cytoskeleton to form a spindle-shaped cell with abundant stress fibres. The fact that EMT enables cells to be motile is utilised by epithelial cancers to achieve invasiveness. EMT can be induced both by environmental factors, such as hypoxia, and by extracellular signalling molecules such as TGF-β. Up-regulation of EMT transcription factors, including Snail, Slug, ZEB1 and ZEB2, leads to the EMT-defining down-regulation of E-cadherin [1], [2]. Although the dramatic increase in F-actin and stress fibres and fibroblast-like morphology are all hallmarks of EMT, the actin nucleators involved in this cytoskeletal remodelling have been poorly characterized.

Formins are highly conserved actin nucleating proteins that are present in all eukaryotic organisms. The formin homology 2 (FH2) domain is the defining element in formins. The flanking regions vary considerably between individual formin proteins, probably resulting in different cellular functions and regulatory mechanisms. Formins catalyse the nucleation of actin at the barbed end of actin filaments. During elongation the dimerised formins stay attached to the filament, thereby protecting it from severing molecules [3]. The activity of formins is regulated by Rho GTPases, molecular switches which remodel the cytoskeleton in different cellular compartments, controlling stress fibre assembly, focal adhesion formation and the mode of motility in cancer cells [4], [5].

As modulators of cell organisation, movement and development, formins are good candidates in the search for actin organisers that enable the profound cytoskeletal changes in EMT. However, the role of formins in EMT is not known in detail. By using an oral squamous cell cancer (SCC) EMT model, we show here that FHOD1, a primarily mesenchymally expressed formin, is specifically upregulated during EMT. In further studies, we show that FHOD1 is expressed also *in vivo* at the invasive front of SCC and that it is required for maintenance of mesenchymal morphology, efficient migration and invasion.

## Materials and Methods

### Cell lines

Oral squamous cell carcinoma (SCC) cell line UT-SCC-43A was derived from a primary gingival tumour of a 75-year-old Caucasian female. The tumour was staged as T_4_N_1_M_0_, and was histologically a grade 2 SCC [6]. UT-SCC-43B was derived from a recurrent tumour from the same patient after radiation therapy and surgery. Cell line 43A-SNA has been generated by transfecting 43A cells with full-length haemagglutinin-tagged cDNA of murine Snail. The three cell lines have been established earlier, and have previously been found to show changes in the epithelial cell differentiation program through different mechanisms of E-cadherin suppression [7]. Prior to establishment of both primary cell lines UT-SCC-43A and UT-SCC-43B for research, the approval of the Joint Committee on Ethics of the University of Turku and Turku University Hospital was obtained as well as written consent from the donor [7]. The telomerase-immortalized human microvascular endothelium cell line (TIME) and human dermal microvascular endothelial cell line (HMEC) were a kind gift from MSc Johannes Keuschnigg (University of Turku, Turku, Finland; cell lines originally from ATCC). Other cell lines were purchased from ATCC and maintained according to the distributor's instructions.

### Transcriptomic microarray data and quantitative real-time-PCR

Gene expression was analysed using the Illumina HumanHT-12 v4 Expression BeadChip at the Finnish Microarray and Sequencing Centre, Turku Center for Biotechnology. Total RNA was extracted from cultured cells using RNeasy Mini kit (Qiagen) according to the manufacturer's protocol and processed to cDNA with cDNA synthesis kit (Applied Biosystems, Foster City, CA). The array-based data on cell lines has been loaded to ArrayExpress (accession number E-MTAB-1420).

TaqMan qRT-PCR was performed with an Applied Biosystems 7900HT instrument (Finnish Microarray and Sequencing Centre). Probes and primers were from Oligomer, Helsinki, Finland. Quantitation was carried out with RQ manager 1.2 software using the ΔΔCT method (Applied Biosystems). Three replicate samples were studied for detection of target mRNA expression and β-actin used as an endogenous control. The quantities were expressed as an n-fold difference relative to the UT-SCC-43A cell line. The results are presented as means ± SD. Statistical analyses were performed using Student's *t*-test and Pearson's correlation coefficient, unless otherwise indicated. Gene specific primers were: DIAPH1 (forward 5′-cagtcaggggcagcattc-3′, reverse 3′-cactgttcttggacaccttgg-5′), FHOD1 (forward 5′- cctcagctgacacctccag-3′, reverse 3′-cagcgcaacctgcttctc-5′), FHOD3 (forward 5′-ggccaggttggaaaggtc-3′, reverse 3′-tctgctgccagtgactcttg-5′), FMNL3 (forward 5′-ccatcgaggacatcatcaca-3′, reverse 3′-ccgagagggtctcagtgg-5′).

### FHOD1 antibodies

A FHOD1 rabbit anti-human polyclonal monospecific antibody was produced by the Human Protein Atlas program (HPA) [8], and is currently available to the public (HPA024468, Atlas Antibodies, Stockholm, Sweden). Further antibody characterisation is described in the Results section. In some experiments, another polyclonal FHOD1 antibody (Millipore, Bedford, MA, USA) was used. The reactivity of the two antibodies was identical in western blotting and immunostainings.

### Western and Northern blotting of human cell lines

Cell lines MDA-MB-231 (breast cancer cells), WM164 (melanoma cells), A431 (squamous cell carcinoma cells), U138MG (glioblastoma cells), HUVEC (human umbilical vein endothelial cells), HMEC (human dermal microvascular endothelial cells), TIME, UT-SCC-43A, UT-SCC-43B and 43A-SNA were harvested and lysed in RIPA buffer supplemented with protease inhibitors. For each experiment, samples were normalized for protein concentration and equal amounts of material in Laemmli sample buffer were subjected to SDS-PAGE and transferred to nitrocellulose filters. For immunoblotting the HPA anti-FHOD1 antibody was incubated overnight at 1∶2000 dilution in BSA/TBS/Tween 0.1%, followed by secondary antibody [HRP-conjugated swine anti-rabbit (Dako, Glostrup, Denmark)]. Bound proteins were detected by enhanced chemiluminescence. In the peptide competition experiment the antibody was first incubated with a 100-fold molar excess of GST-FHOD1 fusion protein containing the antigenic epitopes for 1hour, or with GST as a control. Protein loading was checked by immunoblotting with an α-tubulin antibody (Invitrogen, Carlsbad, CA, USA). Epithelial/mesenchymal transdifferentation was checked by immunoblotting with anti-E-cadherin and anti N-cadherin antibodies (BD Biosciences, San Jose, CA, USA).

Northern blot analysis was performed as described [9]. The sequence of the probe included most of the antibody epitope (NCBI RNA RefSeq clone GenBank NM_013241.2, bases 1517–1810).

### Inhibition of MAPK/ERK and PI3K signalling pathways

UT-SCC-43B cells were plated in complete medium and cultured for 24 hours before medium was replaced by serum free medium and incubated overnight. Next, the cells were incubated for 4 days in 10µM MEK 1/2 inhibitor U0126 (Cell Signaling Technology, Danvers, MA, USA) or PI3 kinase inhibitor LY294002 (Tocris Bioscience, Bristol, UK) in 1% serum medium for U0126 and 10% serum medium for LY 294002. Control cells were treated with the same volume of the vehicle. After incubation, the efficacy of MAPK pathway inhibition was checked by immunoblotting as described above with 1∶1000 dilutions of polyclonal anti-Phospho-p44/42 MAPK (Thr202/Tyr204) (p-ERK 1/2) (Cell Signaling Technology) and polyclonal anti-ERK 2 (MAPK 2) antibody (Santa Cruz Biotechnology, Santa Cruz, CA, USA). PI3K pathway inhibition was checked by immunoblotting with monoclonal anti-Phospho-Akt (Ser473) (p-Akt), monoclonal anti-Akt (pan) (both from Cell Signaling Technology).

### Gene silencing

FHOD1 expression was transiently knocked down in MDA-MB-231, TIME and UT-SCC-43B cells using *SMART*pool siRNA (Dharmacon Research, Boulder, CA). Non-targeting Pool siRNA (Dharmacon) was used as a control. Cells were transfected with Dharmafect 1 (Dharmacon) according to manufacturer's instructions. FHOD1 levels were examined in cell lysates 72 hours after transfection by immunoblotting.

### *In silico* transcriptomics analysis

The GeneSapiens database was utilized to study the FHOD1 mRNA expression across all human normal tissues [10]. The samples included in this database have been analysed on the Affymetrix platform and due to unique normalization and data quality verifications, gene expression profiles collected from different studies can be combined to generate an overview of the expression profile in human tissues.

### Immunohistochemistry

Normal tissues were collected, fixed and immunohistochemically stained as described [9]. The collection of normal tissues for this study was approved by the Joint Committee on Ethics of the University of Turku and Turku University Hospital as well as written consent from the donors. The 10 paraffin embedded oral SCC samples were collected from the tissue archive of the Department of Pathology at Turku University Hospital with the approval of the Joint Committee on Ethics of the University of Turku and Turku University Hospital. According to the Finnish legislation (Law on the use of tissue specimens for research, [11, 20 §]), the permission to use specimens collected for diagnostic purposes, is granted by local institutional authorities in situations, where patient information is not included. Therefore, patients have not been consented, but the permission has been granted by the local ethics committee and the medical director of Turku University Hospital. The HPA FHOD1 antibody was used at a 1∶250 dilution. Individual tissues and cell types were evaluated by scoring the staining intensity by grading from 0 (no staining) to +++ (strong staining).

### Immunofluorescence microscopy and quantification of F-actin

UT-SCC-43A and UT-SCC-43B cells were cultured on gelatine-coated coverslips, and fixed with 4% paraformaldehyde for 15 min. The cells were permeabilised with cold acetone for 4 min. After 30 min in 1% bovine serum albumin (BSA) in PBS, the cells were incubated for 1 h with the FHOD1 antibody (1:250), followed by a secondary antibody, Alexa 568-conjugated goat anti-rabbit (1∶500, Molecular Probes, Eugene, OR, USA). F-actin was visualized with Alexa 488-conjugated phalloidin (Molecular Probes). The mounting media contained DAPI for staining nuclei (Vector Laboratories, Burlingame, CA, USA). Invadopodia were visualised by immunofluorescence staining with anti-cortactin 1∶200 (Millipore). Plasma cells were identified with anti-CD138 mAb (Ventana Medical Systems, Tucson, Arizona, USA). Alexa Fluor 568-conjugated goat anti-mouse was used as secondary antibody (Molecular Probes). The cells were imaged with immunofluorescence microscopy or a Zeiss LSM 510 Meta confocal microscope (Carl Zeiss, 63× Plan Apochromat objective, Göttingen, Germany). Image J 1.42q (NIH, USA) software was used in picture analyses. Quantification of F-actin in UT-SCC-43B cells was done by analysing Alexa 488-conjugated phalloidin staining from confocal images of 20 cells from each experiment. Mean fluorescent intensities were measured from the cell cytoplasm. The significance of the experiment was calculated using Students independent samples *t*-test.

### Wound healing and invasion assays

Migration of UT-SCC-43B cells treated with FHOD1 or non-targeting siRNAs was studied on gelatine-coated 24-well-plates. A wound was created by manually scraping the monolayer of cells with a 10 μl pipette tip. The cells were washed with PBS, and filtered medium was added. An image of cells migrating into the wound was taken at 10 min intervals for 24 h. ImageJ software was used for measuring the wound area at 1 h intervals. The wounds were analysed using repeated measurements analysis of variance (rmANOVA). Time was held as repeated effect and group as fixed effect. Statistical analyses were carried out using SAS system for Windows, Version 9.2 (SAS Institute Inc, Cary, NC, USA). The effect of FHOD1 silencing on invasive capacity of UT-SCC-43B cells was studied using the IncuCyte real-time imaging system (Essen BioScience, Ann Arbor, Michigan, USA). UT-SCC-43B cells treated with FHOD1 siRNA or non-coding siRNA and untreated control cells were plated on 96-well plates (ImageLock plate, Essen BioScience, MI) coated with 50 µl 10% Growth Factor Reduced Matrigel (BD Biosciences) after which the cells were allowed to attach o/n at +37°C. A wound was scratched across each well (Wound Maker, Essen BioScience) and the growth media was removed. The cells were then carefully covered with 50 µl 25% Matrigel in normal growth medium and incubated in 37°C for 2-3 hours to allow gelling, after which 100 µl of growth medium was carefully added to each well. The rate of invasion (wound closure through the matrix) was monitored hourly with Incucyte imaging software (Essen BioScience) for 72 hours. Invasion efficiency was determined as percentage of the relative wound confluence compared to respective negative control (regarded as 100%).

### Measurement of extracellular matrix degradation and invadopodia quantification

To analyse the influence of FHOD1 knockdown on extracellular matrix (ECM) degradation and invadopodia formation of UT-SCC-43B cells, cells were treated with non-coding siRNA and FHOD1 siRNA as described above and plated on 8-well glass slides pre-coated with Cy3-labeled gelatine according to manufacturer's instructions (QCM Gelatine Invadopodia Assay (Red), Millipore). After 24 h incubation at 37°C cells were fixed with 4% paraformaldehyde and stained for immunofluorescence microscopy with anti-cortactin or phalloidin. The ECM degradation was visible as dark foci devoid of fluorescence. Images were acquired with an Olympus BX60 fluorescence microscope (Olympus Microscopes, Essex, UK). Degradation cavities produced by cells were photographed and resorption areas per cell (px) were measured with ImageJ software. For each group, the mean degradation of 100 cells from eight wells was compared. To evaluate invadopodia formation, cells were checked for actin-rich comet- or ring-like protrusions with positive cortactin staining at the ventral surface. The percentage of cells containing invadopodia was counted from 10 different fields in each group. Differences between groups were tested for significance by ANOVA (Tukey post hoc) in both experiments.

## Results

### Transcriptional regulation of formins during cancer-associated EMT

We used three model cell lines to evaluate alterations in formin expression during cancer-associated EMT. UT-SCC-43A is an oral SCC cell line from primary tumour and UT-SCC-43B is a line from the same tumour recurring after surgery and radiotherapy. The recurring tumour has undergone a spontaneous EMT, as demonstrated by several markers [7]. The third cell line, 43A-SNA, was established by transfecting UT-SCC-43A cell with Snail to induce EMT.

**Figure 1:**
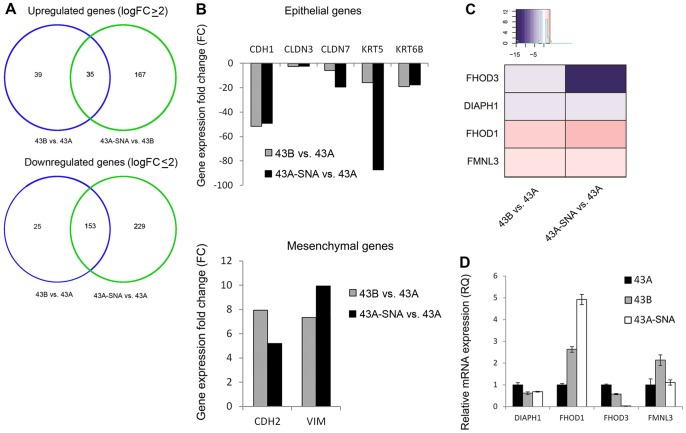
Transcriptomic analysis of the UT-SCC-43A, UT-SCC-43B and 43A-SNA oral SCC cell lines. A) Microarray transcriptomic profiling of UT-SCC-43A, UT-SCC-43B and 43A-SNA cells. Transcripts significantly altered in UT-SCC-43B cells in comparison with UT-SCC-43A cells are indicated by the blue circle and transcripts altered in 43A-SNA cells in comparison with UT-SCC-43A are indicated by the green circle. The number of genes altered in both comparisons is shown in the overlapping area. The number of up-regulated genes is shown on the top and down-regulated genes on the bottom. B) mRNA level alterations for selected epithelial (top) and mesenchymal (bottom) genes. Depicted genes encode following proteins: CDH1 =  E-cadherin, CLDN3 =  Claudin 3, CLDN 7 =  Claudin 7, KRT5 =  Keratin 5, KRT6B  =  Keratin 6b, CDH2 =  N-Cadherin, VIM  =  Vimentin. C) mRNA levels for formins with significant alterations in both UT-SCC-43B and 43A-SNA cells that were subsequently confirmed by RT-PCR. D) mRNA levels for formins confirmed by RT-PCR.

Transcriptomic analysis revealed that the mesenchymal cell lines UT-SCC-43B and 43A-SNA have 35 up-regulated genes (logFC ≥2, p≤0.001) and 153 down-regulated genes (logFC ≤ −2, p≤0.001) in common (Figure 1 A; gene lists presented in Table S1). In UT-SCC43B and 43A-SNA, established EMT-markers such as N-cadherin, vimentin and collagens were up-regulated (Figure 1 B), whereas epithelial markers such as E-cadherin, keratins, integrins and laminins were simultaneously down-regulated indicating that the transcriptomic alterations are consistent with EMT.

Of the 13 formin family members included in the array (DAAM1, DAAM2, DIAPH1, DIAPH2, DIAPH3, FHOD1, FHOD3, FMN1, FMN2, FMNL1, FMNL2, FMNL3, INF2), two were consistently up-regulated and four were down-regulated during EMT. The most significant up-regulation was seen with FHOD1, which was increased 2.3-fold in UT-SCC-43B and 3.2-fold in 43A-SNA as compared with UT-SCC-43A (Figure 1 C). The significant transcriptional differences were further verified by qRT-PCR, which demonstrated upregulation of formins FHOD1 and FMNL3 and downregulation of FHOD3 and DIAPH1 in both UT-SCC-43B and 43A-SNA cells (Figure 1 D).

### Characterization of human FHOD1 and its expression pattern

As FHOD1 was the most up-regulated formin in EMT, we decided to further study this poorly characterised protein. The expression profile of human FHOD1 is not known, largely due to lack of suitable antibodies. We took advantage of a FHOD1 antibody raised by the Human Protein Atlas project. In Western blotting, the antibody reacted with several human endothelial and cancer cell lines. A single band of 145 kDa, which is slightly larger than the predicted 127 kDa, was detected in all tested cell lines (Figure S1 A). A similar band was also seen with another FHOD1 antibody. The wide reactivity is in line with earlier findings demonstrating FHOD1 expression in most immortalized cell lines [11].

To further test the specificity of the HPA antibody, the reactivity was blocked with the antigenic GST-FHOD1 fragment or GST. As FHOD1 is considered to be an endothelial formin [12], we used lysates from three endothelial cell lines: HMEC, TIME and HUVEC. Incubation with the GST-FHOD1-peptide virtually abolished detection of the 145 kDa bands in all cell lines (Figure S1 B), whereas incubation with GST did not have any effect. Finally, we confirmed the specificity by transfecting MDA-MB-231 cells with FHOD1 small interfering (si) RNA or with non-targeting control siRNA. After FHOD1 siRNA treatment, the antibody reactivity was significantly reduced whereas the reactivity in cells transfected with control siRNA was unaffected (Figure S1 C).

The FHOD1 transcript in cell lines was analysed by Northern blotting. The analysis revealed a single 4.0 kb transcript in all five cell lines, matching the result from the Western blot analysis (Figure S1 D).

**Figure 2:**
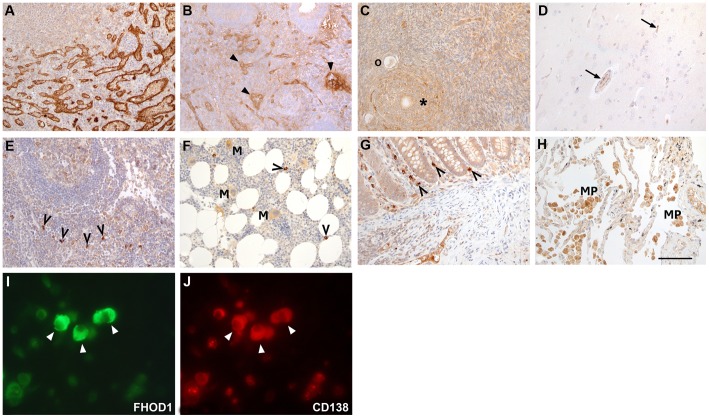
FHOD1 is strongly expressed in endothelium and plasma cells but only weakly in epithelial cells. Paraffin-embedded normal human tissues were stained with FHOD1 antibody. A) In the spleen, endothelial cells lining blood vessels show intensive FHOD1 immunoreactivity. B) Endometrial stroma and glands express little FHOD1. Endothelial cells in blood vessel walls are stained strongly (arrowheads). C) In the ovary, oocytes (o) and follicle cells (asterisk) express little FHOD1. D) In the brain, neither neurones nor glial cells show FHOD1 staining. The endothelial cells in minute capillaries (arrows) stain weakly. E) In the lymph node, most lymphocytes stain weakly. Occasional strongly staining cells have plasma cell morphology (open arrowheads). F) In the bone marrow, erythropoietic cells do not express FHOD1. Myelopoietic cells and megakaryocytes (M) stain weakly. Occasional small cells that express FHOD1 strongly (open arrowheads) are plasma cells. G) In colonic mucosa, epithelial cells show only weak FHOD1 staining. Stromal plasma cells (open arrowheads) express FHOD1 strongly. H) Alveolar epithelium in the lung stains weakly for FHOD1, while alveolar macrophages (MP) show moderate FHOD1 staining. I, J) Double immunofluorescence staining of FHOD1-positive inflammatory cells demonstrates that the same cells express CD138 (arrowheads). The cell morphology and CD138 indicate that they are plasma cells. In A–H scale bar  = 100 μm.

To systematically investigate which tissues and cell types express FHOD1, immunohistochemical staining using the HPA FHOD1 antibody was performed in 26 different human tissues. In virtually all samples capillary endothelial cells were immunoreactive, although with variable staining intensity. The most intensive FHOD1 staining was seen in the small blood vessels of the spleen (Figure 2 A), endometrium (Figure 2 B), ovary (Figure 2 C) and in peritoneal vessels below the mesothelium. In most tissues and cell types, the parenchymal cells demonstrated little or no FHOD1 reactivity. For instance, in the brain, neither nerve cells nor glial cells expressed FHOD1 (Figure 2 D). Endothelial cells in cerebral capillaries, on the other hand, expressed FHOD1. A small subset of lymphocytes in lymph nodes (Figure 2 E), spleen, bone marrow (Figure 2 F) and intestinal mucosa (Figure 2 G) stained intensely. In the lung, moderate expression was seen in alveolar macrophages (Figure 2 H). The results of the immunohistochemical analysis are presented in Table 1. Subsequent staining with lymphocyte subtype-specific markers revealed that the subset of lymphocytes that expressed FHOD1 consisted of CD138-positive plasma cells (Figures 2 I and J).

**Table 1:** Expression of FHOD1 in various tissues and cell types.

Tissue	Cell type	Staining*	Comments
**Adrenal gland**	Cortical cells	+	
	Medullary cells	+	
**Bone marrow**	Erythroid cells	0	
	Myeloid cells	+	
	Megakaryocytes	+	
**Brain**	Neurons	0	
	Glial cells	0	
**Breast**	Ductal epithelium	+	
	Lobular epithelium	+	
	Myoepithelium	+	
**Colon**	Epithelium	+	
**Duodenum**	Enterocytes	+	
	Brunner glands	0	
	Stromal cells	0	Plasma cells +++
**Kidney**	Podocytes	0	
	Mesangial cells	0	
	Tubular epithelium	0	
**Liver**	Hepatocytes	0	
	Biliary duct epithelium	0	
**Lung**	Alveolar epithelium	+	
	Alveolar macrophages	++	
**Lymph node**	Follicular cells	+	
	Paracortical cells	+	Plasma cells +++
**Mouth**	Squamous epithelium	0	
**Oesophagus**	Squamous epithelium	0	
**Ovary**	Follicle cells	+	
	Stromal cells	+	
**Pancreas**	Exocrine epithelium	0	
	Ductal epithelium	0	
	Endocrine cells	0	Endothelium ++
**Parotid gland**	Acinar cells	0	endothelium +
	Adipocytes	0	
**Placenta**	Trophoblast	0	
	Stromal cells	0	
	Endothelium	++	
**Prostate**	Epithelium	0	
	Smooth muscle cells	0	
	Peripheral nerve cells	0	
**Skin**	Keratinocytes	0	Minority of basal cells +
**Small intestine**	Enterocytes	+	
**Spleen**	Endothelium	+++	
	White pulp cells	0	
	Red pulp cells	0	
**Stomach**	Glandular cells	+	
	Stromal cells	0	
**Striated muscle**	Myocytes	+	
**Testis**	Spermatocytes	0	
	Sertoli cells	0	
	Leydig cells	+	
**Thyroid gland**	Epithelium	+	
**Urinary bladder**	Urothelial cells	+	Endothelium +
**Uterus**	Smooth muscle cells	0	Endothelium +++
	Glandular cells	+	
	Endometrial stromal cells	+	

* 0 =  no staining, + =  weak staining, ++ =  moderate staining, +++ =  strong staining.

We conclude that FHOD1 is expressed in many tissues but in only restricted cell types. Of note, all of the strongly staining cell types are of mesenchymal origin. This matches the *in silico* mRNA profile obtained from a GeneSapiens database search [10] (Figure S2). The low ubiquitous FHOD1 expression across all normal tissues could be attributable to expression in the endothelial cells. Higher expression can be seen in tissues where parenchymal cells also stained in immunohistochemistry, *i.e.* skeletal muscle, breast, and ovary. Although the mRNA level in mesothelial tissue samples is relatively high, mesothelial cells did not stain with the FHOD1 antibody. The high mRNA levels are probably due to the abundant endothelium in capillaries below the mesothelium. In such vessels, immunohistochemical FHOD1 staining was strong.

### EMT leads to PI3K pathway-dependent FHOD1 upregulation and morphological alterations

**Figure 3:**
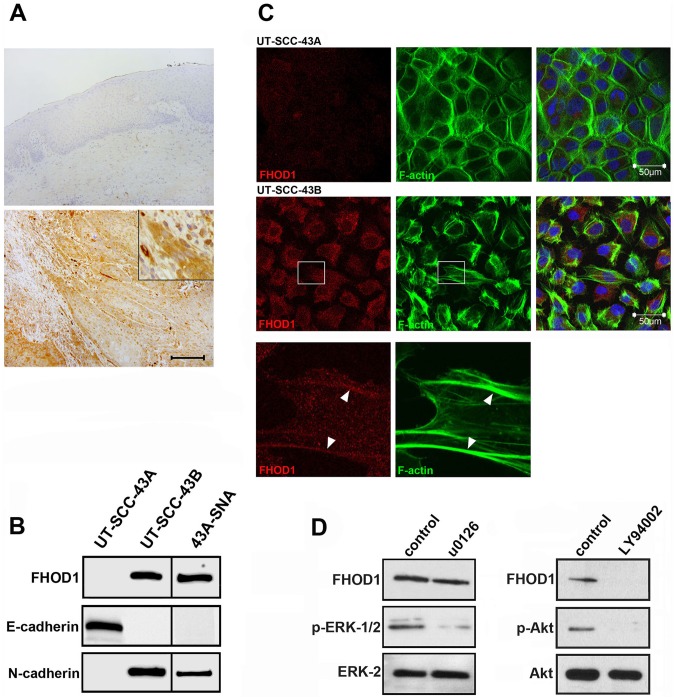
FHOD1 is upregulated in clinical oral SCC, as well as *in vitro* in SCC cells with EMT features. A) In normal or non-neoplastic stratified squamous epithelium, no FHOD1 can be detected (top). In invasive squamous cell carcinomas, moderate to strong FHOD1 immunoreactivity is seen in spindle-shaped cells at the invasive front that on morphological grounds have undergone EMT (bottom; details in inset). In the tumour bulk, which consists of cells with an epithelial morphology, only weak immunoreactivity is present. Scale bar: 200 μm. B) Western blot analysis of cell lines show that the epithelial SCC cell line UT-SCC-43A does not express detectable FHOD1, while both the spontaneous EMT cell line UT-SCC-43B and the Snail-induced EMT cell line 43A-SNA express FHOD1. UT-SCC-43A expresses the epithelial marker E-cadherin but not N-cadherin, whereas UT-SCC43-B and 43A-SNA express N-cadherin but not E-cadherin. C) F-actin organization of UT-SCC-43A (upper panel) is typically epithelial, with distinct cell submembraneous filaments and scant stress fibres. UT-SCC-43B shows features of mesenchymal organization (middle panel). Cell-cell contacts are few, the cells are elongated and contain lamellopodia, filopodia and stress fibres. The insert (bottom panel) shows that a fraction of FHOD1 co-localizes with stress fibres in UT-SCC-43B cells (arrowheads). Nuclei are stained with DAPI (blue). D) FHOD1 upregulation in UT-SCC-43B cells is dependent of PI3K signalling. Treatment with MEK 1/2 inhibitor U0126 reduces phosphorylation of ERK 1/2 but does not influence FHOD1 expression. In contrast, PI3K inhibition by LY294992 markedly reduces FHOD1 expression. The reduction of p-Akt indicates that the pathway is efficiently inhibited.

Although the immunohistochemical results and in *silico* mRNA profile suggested minimal FHOD1 expression in epithelial cell types, our transcriptomics results and GeneSapiens bioinformatics survey of carcinomas (not shown) indicated moderate mRNA levels in certain cancers. For instance, the mean normalised expression value of lung adenocarcinoma was 850 as compared to 400 in the respiratory system, and the value of oral SCC was 800 as compared to 100 in normal skin (values for oral mucosa were not available). To investigate whether the upregulation of FHOD1 occurs in clinical oral SCC, we randomly chose ten oral SCC specimens for immunohistochemical analysis. As EMT is thought to occur *in vivo* at the invasive edge of epithelial cancers, tissue microarrays could not be used. We found no reactivity in the non-neoplastic stratified squamous epithelium of oral mucosa in any of the cases, while moderate to strong FHOD1 staining was consistently seen in the invasive SCC cells with a mesenchymal spindle-shaped morphology (Figure 3 A). Interestingly, FHOD1 immunoreactivity was mild or absent in the well-differentiated areas of invasive cancer, indicating that the tumour bulk that consists of cellular areas with epithelial differentiation expresses little FHOD1.

Next, we studied whether FHOD1 expression was associated with morphological and functional alterations in EMT. Of the three oral SCC cell lines, detectable FHOD1 was seen in UT-SCC-43B and 43A-SNA but not in UT-SCC-43A, indicating that also at protein level, FHOD1 up-regulation occurs in EMT both in the spontaneous and Snail-induced model (Figure 3 B). As expected, UT-SCC-43A expressed E-cadherin but not N-cadherin, while UT-SCC-43B and 43A-SNA expressed N-cadherin but no E-cadherin (Figure 3 B).

The cell lines also showed distinct morphological features and organisation of actin cytoskeleton. UT-SCC-43A cells had a typical epithelial phenotype, as they formed sheets of cells with cell-to-cell contacts and scarce stress fibres (Figure 3 C, upper panel). UT-SCC-43B, on the other hand, consisted of slightly elongated cells with many filopodia and abundant stress fibres (Figure 3 C, middle panel). The distribution of FHOD1 was in part punctuate and cytoplasmic, but it also clearly localised to stress fibres (Figure 3 C, lower panel).

In oral SCC, signalling pathways commonly activated in EMT include the Phosphatidylinositol-s-Kinase (PI3K) and Mitogen-activated protein kinase (MAPK/ERK 1/2) pathways [13]. To investigate whether FHOD1 upregulation was dependent on activation of either of these signalling pathways, the cells were treated with PI3K and MEK1/2 inhibitors. The inhibition of MEK1/2 did not influence FHOD1 expression, although the pathway was efficiently inhibited. In contrast, PI3K inhibition substantially reduced FHOD1 expression (Figure 3 D). This finding indicates that FHOD1 expression in UT-SCC-43B is dependent of PI3K signalling.

### FHOD1 knockdown represses mesenchymal morphology, migration and invasion in UT-SCC-43B cells

**Figure 4:**
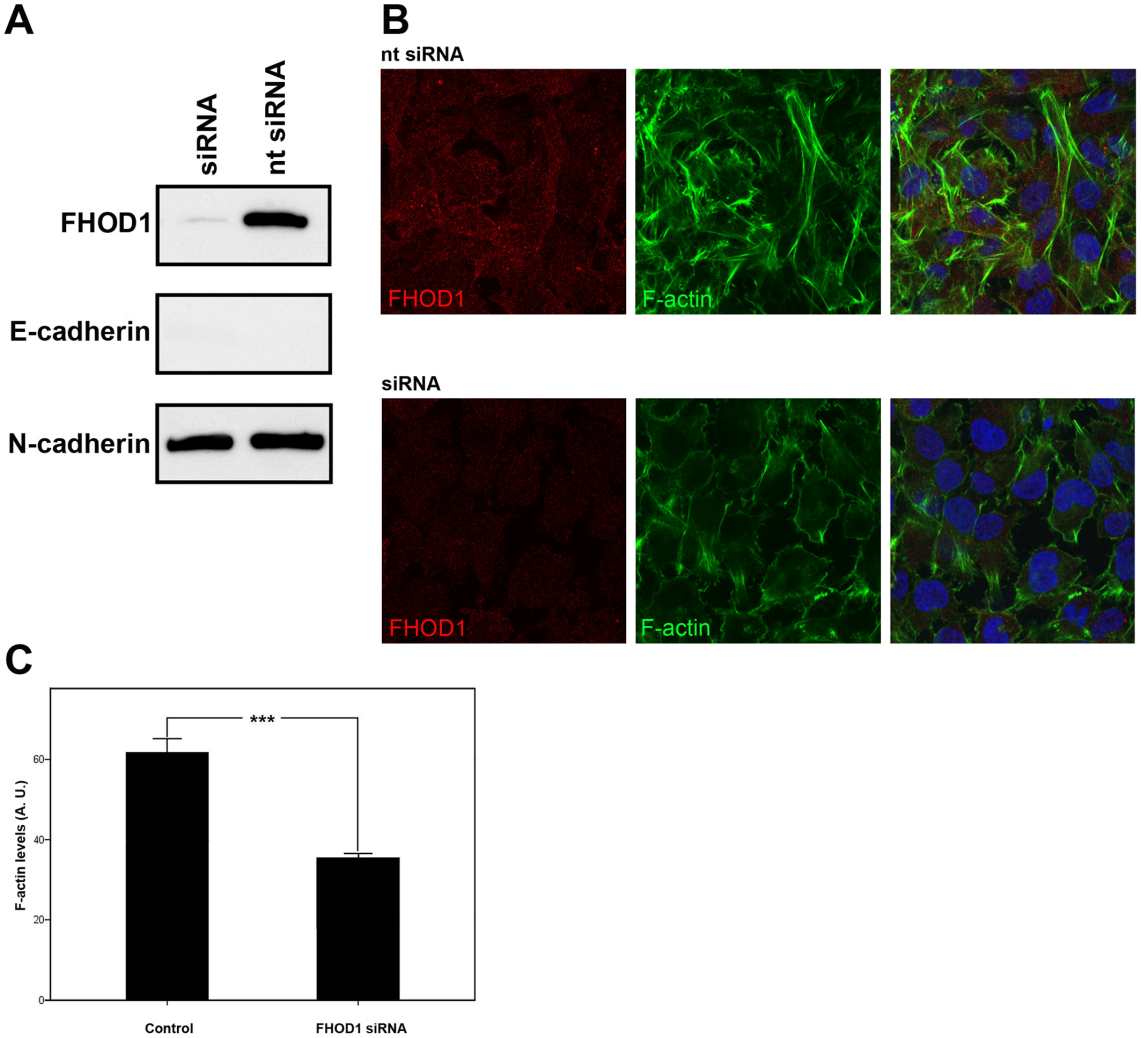
FHOD1 siRNA knockdown changes the morphology of UT-SCC-43B cells and reduces the number of stress fibres. A) Western blotting shows that FHOD1 siRNA treatment significantly reduces FHOD1 expression. The cells are still E-cadherin negative and the expression of N-cadherin is unaltered. B) Cells treated with non-targeting siRNA (upper panel) express FHOD1 and have a mesenchymal phenotype. Stress fibres are abundant. FHOD1 siRNA abolishes FHOD1 staining (lower panel). siRNA treated cells have less actin stress fibres and are morphologically rounder and flatter. Nuclei are stained with DAPI (blue). C) F-actin staining is significantly reduced in FHOD1 siRNA treated cells. Phalloidin staining intensity is reduced by 49%. Bars indicate standard error of mean. *** p<0.0001. AU  =  arbitrary units.

The functional significance of FHOD1 expression in the EMT cell line UT-SCC-43B was studied by RNA interference, which achieved a significant decrease in FHOD1 expression. E-cadherin and N-cadherin levels were unaffected (Figure 4 A). The knock-down cells were less elongated than control cells and had less actin stress fibres (p<0.0001) (Figure 4 B), indicating a role for FHOD1 in mesenchymal morphology. Quantitative analysis of cytoplasmic phalloidin staining confirmed the reduced F-actin content in cells treated with FHOD1 siRNA (Figure 4 C).

**Figure 5:**
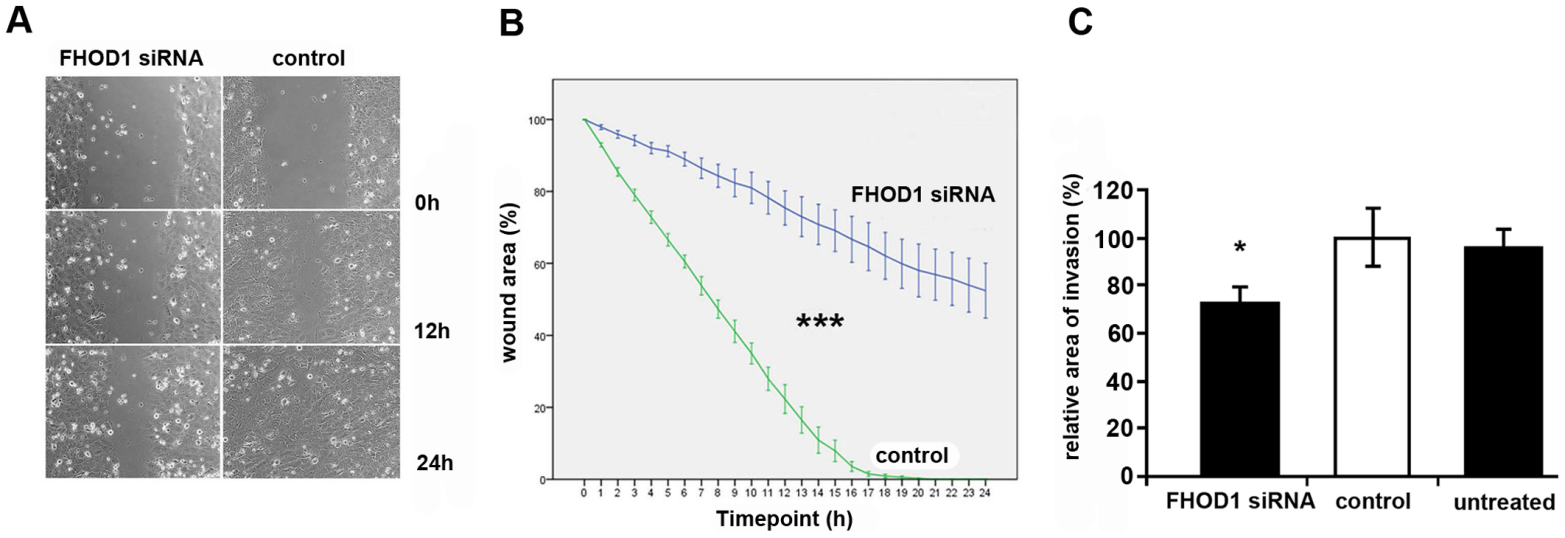
FHOD1 silencing significantly reduces the motility and invasiveness of UT-SCC-43B cells. UT-SCC-43B cells were treated with FHOD1 or control siRNA and subjected to wound healing assay and a Matrigel invasion assay. A) The wound of control cells has fully healed by 24 h, whereas the wound in siRNA treated cells is only partially healed. B) A graph showing the healing process at different time points. Bars indicate standard error of mean. Interaction between time and group are statistically significant (*** P<0.0001). The difference is statistically significant at every time point (P<0.002). n = 4 in each group. C) In a 72 hour Matrigel invasion assay, invasion is significantly reduced in FHOD1-deficient cells when compared to control siRNA treated cells. (* P<0.05) n = 3–5 in each group.

In order to assess whether the decrease in actin fibres would correlate with changes in motility, cells were subjected to a wound healing assay. In this assay, siRNA treated cells migrated slowly and failed to heal the wound in 24 hours, while control cells healed the wound within 18 hours (Figure 5 A). Both siRNA treated and control cells formed lamellipodia, but the active forward movement of the cell body was markedly reduced in siRNA cells. The difference in wound healing efficacy was statistically significant already at one hour and remained so at every time point (p<0.002) (Figure 5 B). The interaction between time and groups was statistically significant (p<0.0001).

The influence of FHOD1 knockdown on invasiveness was analysed using the IncuCyte live cell imaging system with Matrigel as a 3D environment. FHOD1-depleted cells invaded less efficiently than control cells (p<0.05), indicating that FHOD1 is relevant not only for migration in 2D conditions but also for invasive capacity of cancer cells (Figure 5 C).

### FHOD1 silencing affects invadopodia formation and proteolysis

**Figure 6:**
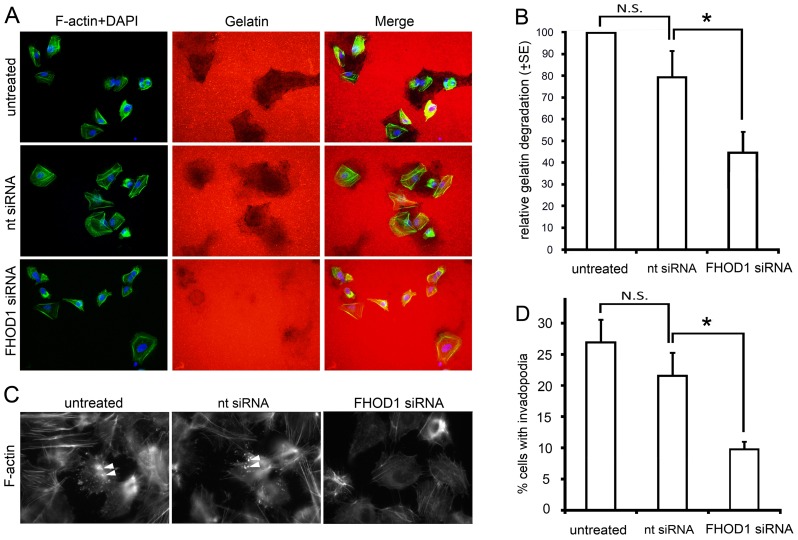
FHOD1 silencing inhibits proteolytic activity and invadopodia formation. A) Images from a zymography assay performed with untreated, nt siRNA and FHOD1 siRNA treated SCC-43B cells. Degradation of Cy 3 labelled gelatin is reduced in FHOD1 siRNA treated cells. B) Quantification of the degraded area/cell. C) Invadopodia formation is visualized by phalloidin staining, which reveals the comet- or ring-shaped actin structures on the ventral cell surface (arrowheads). Invadopodia are present in a larger proportion of untreated and control cells than in FHOD1 siRNA treated cells. D) Quantification of percentage of cells with invadopodia. (* P<0.05). N.S.  =  not significant.

Invasiveness is achieved not only by cytoskeletal alterations, but also by protein degradation which takes place at the tips of invadopodia. To assess whether FHOD1 contributes to protein degradation efficacy, a fluorescent gelatine degradation experiment was conducted with UT-SCC-43B cells transiently silenced for FHOD1 expression. After 24 hours, FHOD1 silenced cells had degraded significantly smaller areas of Cy3 labelled gelatine, as compared to control cells or cells transfected with non-targeting siRNA (p<0.05; Figure 6 A, B). Invadopodia formation was evaluated by counting the proportion of cells with distinct phalloidin and cortactin-containing comet- or ring-like structures on the ventral cell surface (Figure 6 C). Invadopodia-containing cells were significantly fewer in FHOD1 depleted cells than in control cells or cells transfected with non-targeting siRNA (p<0.05; Fig 6 D).

## Discussion

A characteristic feature of cancer-associated EMT is profound rearrangement of the actin cytoskeleton. With this change, cancer cells achieve a migratory and invasive phenotype for crossing tissue barriers and thereby reaching blood and lymphatic vessels. The actin modulators responsible for this change are poorly understood. Here, we show that the formin family member FHOD1 is upregulated in squamous cell cancer EMT and influences the morphologic and functional hallmarks of EMT: actin organisation, cell migration and ability to degrade the extracellular matrix.

The increase of FHOD1 expression appears to be induced by at least two EMT-triggering transcription factors and to occur both *in vitro* and *in vivo*. The EMT phenotype of UT-SCC-43B is related to increased ZEB1 and ZEB2 expression [7], while EMT of the 43A-SNA cell line is induced by the transcription factor Snail. In both EMT cell lines, FHOD1 upregulation was notable both at transcriptional and protein level. In the clinical setting, FHOD1 upregulation was seen in oral SCC samples. Areas of moderate to high immunoreactivity were seen in all cases, in contrast to normal squamous epithelium, where the expression was below detection level. Importantly, the highest FHOD1 expression was seen in areas, where cancer-related EMT is thought to occur *in vivo*, *i.e*. at the invasive front in elongated spindle-shaped tumour cells. So far, the role of formins in cancer-associated EMT has remained elusive, apart from a single report demonstrating an association between FMNL2 and colorectal cancer EMT [14]. In our SCC cell lines, FMNL2 expression was not altered suggesting a tumour/tissue specific control of formin expression in EMT.

FHOD1 functions have previously been studied mainly in mesenchymal cell types, such as fibroblasts and endothelial cells [15], [16]. Our FHOD1 expression profiling, both at tissue and transcriptomic level, supports the view that FHOD1 functions, indeed, are related to mesenchymal rather than epithelial cells. The role of FHOD1 in epithelially derived cancer cells has previously been addressed in one publication [17]. In this recent study, FHOD1 was shown to promote mesenchymal phenotype and invasion of breast cancer cells. In melanoma cells, overexpression of FHOD1 has been found to cause an elongated phenotype and increased migration [18]. Our migration and invasion study, in which we depleted FHOD1 from the EMT cell line UT-SCC-43B, supports the results from both previous studies, since FHOD1 silencing in mesenchymally transformed SCC cells significantly reduced migration and invasion. Taken together, these studies suggest that FHOD1 may mediate cytoskeletal changes and migratory properties in cancer-associated EMT of versatile cancer types.

The activity of FHOD1 is regulated by an intrinsic conformational autoinhibition [19], and activation is required for its ability to induce accumulation of actin stress fibres and co-localise with them [15], [20]. FHOD1 is activated by Rho-associated kinase serine/threonine kinase (ROCK) by phosphorylation of three C-terminal residues of FHOD1, which releases the autoinhibition [16]. ROCK, in turn, is activated by the RhoGTPase RhoA. Activation of the Rho-ROCK cascade is known to induce stress fibres. In line with being downstream of these regulators, the major morphological alterations in FHOD1 depleted cells included reduced formation of actin stress fibres together with loss of the mesenchymal elongated cell shape. Although down-regulation of FHOD1 resulted in a more epithelial phenotype, we did not see a switch from N-Cadherin to E-Cadherin expression during the timeframe of the study. This indicates that the effects of FHOD1 on the actin cytoskeleton are likely to be direct, not mediated by E-cadherin loss. Although we did not address the activity of ROCK in this study, the upregulation of its effector FHOD1 raises a potential treatment promise. ROCK is a known promoter of invasion and metastasis. There are several ROCK-inhibitors that might be effective in distinct types of cancer [21]. Since widespread mesenchymal transformation in head and neck SCC may predict treatment failure and poor outcome, new treatment options would be more than welcome [22].

Invadopodia, actin-rich short-lived membrane protrusions in cancer cells, are reminiscent of their more structured and stable counterpart in benign cells, the podosomes. Invadopodia are crucial for degradation of extracellular matrix (ECM), as they serve as sites for activation and secretion of matrix metalloproteinases (MMPs). The proteolytic activity of MMPs contributes to invasive migration [23]. Invadopodia have been identified in several cancer types, including SCC [24]. There is growing evidence that invadopodia formation is a central feature of EMT-driven invasion [25]–[27]. Whether formins are involved in the formation of invadopodia has remained an unanswered question. Our results demonstrate for the first time that a formin is involved in both degradation of ECM and the formation of invadopodia. However, it seems that this regulation is indirect, since FHOD1 appeared not to be a component of the invadopodial structures. Implicating clinical relevance, increased FHOD1expression in cancer specimens was not global but restricted to cells with mesenchymal morphology at the invasive front.

In conclusion, we recognize FHOD1 as the major formin upregulated in the process of EMT in oral SCC. FHOD1 regulates the alteration of cytoskeletal and functional properties EMT; stress-fibre rich phenotype, efficient migration, proteolysis and invadopodia formation. Importantly, this increase of FHOD1 expression is seen in clinical cancers, in which EMT contributes to dissemination of tumours and subsequent treatment failure. In this setting, the activation cascade of FHOD1 could serve as a potential drug target. Whether increased expression of FHOD1 occurs in SCC in other locations or other types of cancer in general, is an important question that remains to be addressed.

## Supporting Information

Figure S1 **Expression of FHOD1 in cell lines and characterization of the FHOD1 antibody.** A) Western blotting of lysates from four cancer cell lines and TIME endothelial cells. A single band of approximately 145 kDa is detected in all lanes. B) Preincubation of the FHOD1 antibody with a GST-FHOD1′ fusion peptide abrogates the reactivity as compared to incubation with GST alone. C) Transfection of MDA-MB-231 (left panel) or TIME (right panel) cells with FHOD1 siRNA markedly reduces the reactivity, whereas control non-targeting (nt) siRNA has no effect. D) A Northern blot analysis of the same cell lines as in a) shows a single FHOD1 4.0 kb mRNA transcript in the studied cell lines, matching the western blot result.
(JPG)

Figure S2 **FHOD1 mRNA expression profile in normal human tissues.** The expression of FHOD1 mRNA was evaluated using the GeneSapiens database information. The normalized expression values (y-axis) of FHOD1 across normal tissues (x-axis) are presented as box-plots. The box extends from the first to the third quartile of the data and the median is indicated with green. The whiskers extend to the extreme values unless there are outliers. The data observations that lie more than 1.5 * interquartile range (IQR) lower than the first quartile, or 1.5 * IQR higher than the third quartile are considered as outliers and indicated separately. Low FHOD1 expression is seen in most tissues. The highest expression levels are seen in skeletal muscle and mesothelial samples.
(TIF)

Table S1 **Gene lists utilized in the Venn diagram of** **Figure 1A** **.**
(XLSX)

## References

[1] Kalluri R, Weinberg RA (2009) The basics of epithelial-mesenchymal transition. J Clin Invest 119: 1420–1428. 19487818 10.1172/JCI39104 PMC2689101

[2] Iwatsuki M, Mimori K, Yokobori T, Ishi H, Beppu T, et al (2010) Epithelial-mesenchymal transition in cancer development and its clinical significance. Cancer Sci 101: 293–299. 19961486 10.1111/j.1349-7006.2009.01419.x PMC11159985

[3] Faix J, Grosse Rm (2006) Staying in shape with formins. Dev Cell 10: 693–706. 16740473 10.1016/j.devcel.2006.05.001

[4] Schönichen A, Geyer M (2010) Fifteen formins for an actin filament: A molecular view on the regulation of human formins. Bioch Biophys Acta 1803: 152–163. 10.1016/j.bbamcr.2010.01.014 20102729

[5] Parri M, Chiarugi P (2010) Rac and Rho GTPases in cancer cell motility control. Cell Commun Signal 8: 23. 20822528 10.1186/1478-811X-8-23 PMC2941746

[6] Haikonen J, Rantanen V, Pekkola K, Kulmala J, Grénman R (2003) Does skin fibroblast radiosensitivity predict squamous cancer cell radiosensitivity of the same individual? Int J Cancer 103: 784–788. 12516099 10.1002/ijc.10890

[7] Takkunen M, Grenman R, Hukkanen M, Korhonen M, García de Herreros A, et al (2006) Snail-dependent and –independent Epithelial-Mesenchymal Transition in Oral Squamous Carcinoma Cells. J Histochem Cytochem 11: 1263–1275. 10.1369/jhc.6A6958.2006 16899764

[8] Berglund L, Björling E, Oksvold P, Fagerberg L, Asplund A, et al. (2008) A genecentric Human Protein Atlas for expression profiles based on antibodies. Mol Cell Proteomics 7: 2019–2027. Available at: http://www.proteinatlas.org. 10.1074/mcp.R800013-MCP200 18669619

[9] Gardberg M, Talvinen K, Kaipio K, Iljin K, Kampf C, et al (2010) The characterization of diaphanous-related formin FMNL2 in human tissues. BMC Cell Biol 11: 55. 20633255 10.1186/1471-2121-11-55 PMC2912821

[10] Kilpinen S, Autio R, Ojala K, Iljin K, Bucher E, et al. (2008) Systematic bioinformatic analysis of expression levels of 17,330 human genes across 9,783 samples from 175 types of healthy and pathological tissues. Gen Biol 9: R139. Genesapiens website is available at: http://www.genesapiens.com. Accessed 2011 Sep 10. 10.1186/gb-2008-9-9-r139 PMC2592717 18803840

[11] Gill MB, Roecklein-Canfield J, Sage DR, Zambela-Soediono M, Longtine N, et al (2004) EBV attachment stimulates FHOS/FHOD1 redistribution and co-aggregation with CD21: formin interactions with the cytoplasmic domain of CD21. J Cell Sci 117: 2709–2720. 15138285 10.1242/jcs.01113

[12] Wang Y, El-Zaru MR, Surks HK, Mendelsohn ME (2004) Formin Homology Domain Protein (FHOD1) is a cyclic GMP-dependent protein kinase I-binding protein and substrate in vascular smooth muscle cells. J Biol Chem 279: 24420–24426. 15051728 10.1074/jbc.M313823200

[13] Krisanaprakornkit S, Iamaroon A (2012) Epithelial-Mesenchymal Transition in Oral Squamous Cell Carcinoma. Oncology 2012: 681469. 10.5402/2012/681469 PMC3324906 22548191

[14] Li Y, Zhu X, Zeng Y, Wang J, Zhang X, et al (2010) FMNL2 Enhances Invasion of Colorectal Carcinoma by Inducing Epithelial-Mesenchymal transition. Mol Cancer Res 8: 1579–1590. 21071512 10.1158/1541-7786.MCR-10-0081

[15] Gasteier JE, Madrid R, Krautkrämer E, Schröder S, Muranyi W, et al (2003) Activation of the Rac-binding Partner FHOD1 Induces Actin Stress Fibers via a ROCK-dependent Mechanism. J Biol Chem 278: 38902–38912. 12857739 10.1074/jbc.M306229200

[16] Takeya R, Taniguchi K, Narumiya, Sumimoto H (2008) The mammalian formin FHOD1 is activated through phosphorylation by ROCK and mediates thrombin-induced stress fibre formation in endothelial cells. EMBO J 27: 618–628. 18239683 10.1038/emboj.2008.7 PMC2262041

[17] Jurmeister S, Baumann M, Balwierz A, Keklikoglou I, Ward A, et al (2012) MicroRNA-200c Represses Migration and Invasion of Breast Cancer cells by Targeting Actin-Regulatory Proteins FHOD1 and PPM1F. Mol Cell Biol 32: 633–651. 22144583 10.1128/MCB.06212-11 PMC3266604

[18] Koka S, Neudauer CL, Li X, Lewis RE, McCarthy JB, et al (2003) The formin-homology-domain-containing protein FHOD1 enhances cell migration. J Cell Sci 116: 1745–1755. 12665555 10.1242/jcs.00386

[19] Schönichen A, Alexander M, Gasteier JE, Cuesta FE, Fackler OT, et al (2006) Biochemical characterization of the diaphanous autoregulatory interaction in the formin homology protein FHOD1. J Biol Chem 281: 5084–5093. 16361249 10.1074/jbc.M509226200

[20] Schönichen A, Mannherz HG, Behrmann E, Mazur AJ, Kühn S, et al.. (2013) FHOD1 is a combined actin filament capping and bundling factor that selectively associates with actin arcs and stress fibers. J Cell Sci (in press). 10.1242/jcs.126706 23444374

[21] Rath N, Olson MF (2012) Rho-associated kinases in tumorigenesis: re-considering ROCK inhibition for cancer therapy. EMBO Rep 13: 900–908. 22964758 10.1038/embor.2012.127 PMC3463970

[22] Chen C, Zimmermann M, Tinhofer I, Kaufman AM, Albers AE (2012) Epithelial-to-mesenchymal transition and cancer stem (-like) cells in head and neck squamous cell carcinoma. Cancer Lett (in press). 10.1016/j.canlet.2012.06.013 22771535

[23] Sibony-Benyamini H, Gil-Henn H (2012) Invadopodia: The leading force. Eur J Cell Biol 91: 896–901. 22633185 10.1016/j.ejcb.2012.04.001

[24] Hwang YS, Park K, Chung W (2012) Invadopodia formation in oral squamous cell carcinoma: The role of epidermal growth factor receptor signalling. Arch Oral Biol 57: 335–343. 21920495 10.1016/j.archoralbio.2011.08.019

[25] Pignatelli J, Tumbarello DA, Schmidt RP, Turner CE (2012) Hic-5 promotes invadopodia formation and invasion during TGF-β-induced epithelial-mesenchymal transition. J Cell Biol 197: 421–437. 22529104 10.1083/jcb.201108143 PMC3341156

[26] Eckert MA, Lwin TM, Chang AT, Kim J, Danis E, et al (2011) Twist1-induced invadopodia formation promotes tumor metastasis. Cancer Cell 19: 372–386. 21397860 10.1016/j.ccr.2011.01.036 PMC3072410

[27] Takkunen M, Hukkanen M, Liljeström M, Grénman R, Virtanen I (2010) Podosome-like structures of non-invasive carcinoma cells are replaced in epithelial-mesenchymal transition by actin comet-embedded invadopodia. J Cell Mol Med 14: 1569–1593. 19656240 10.1111/j.1582-4934.2009.00868.x PMC3829022

