# Supplementary figures and images for: FHOD1, a Formin Upregulated in Epithelial-Mesenchymal Transition, Participates in Cancer Cell Migration and Invasion
Source: PLoS One. 2013 Sep 26;8(9):e74923. doi: 10.1371/journal.pone.0074923 (PMC3784416; doi:10.1371/journal.pone.0074923)

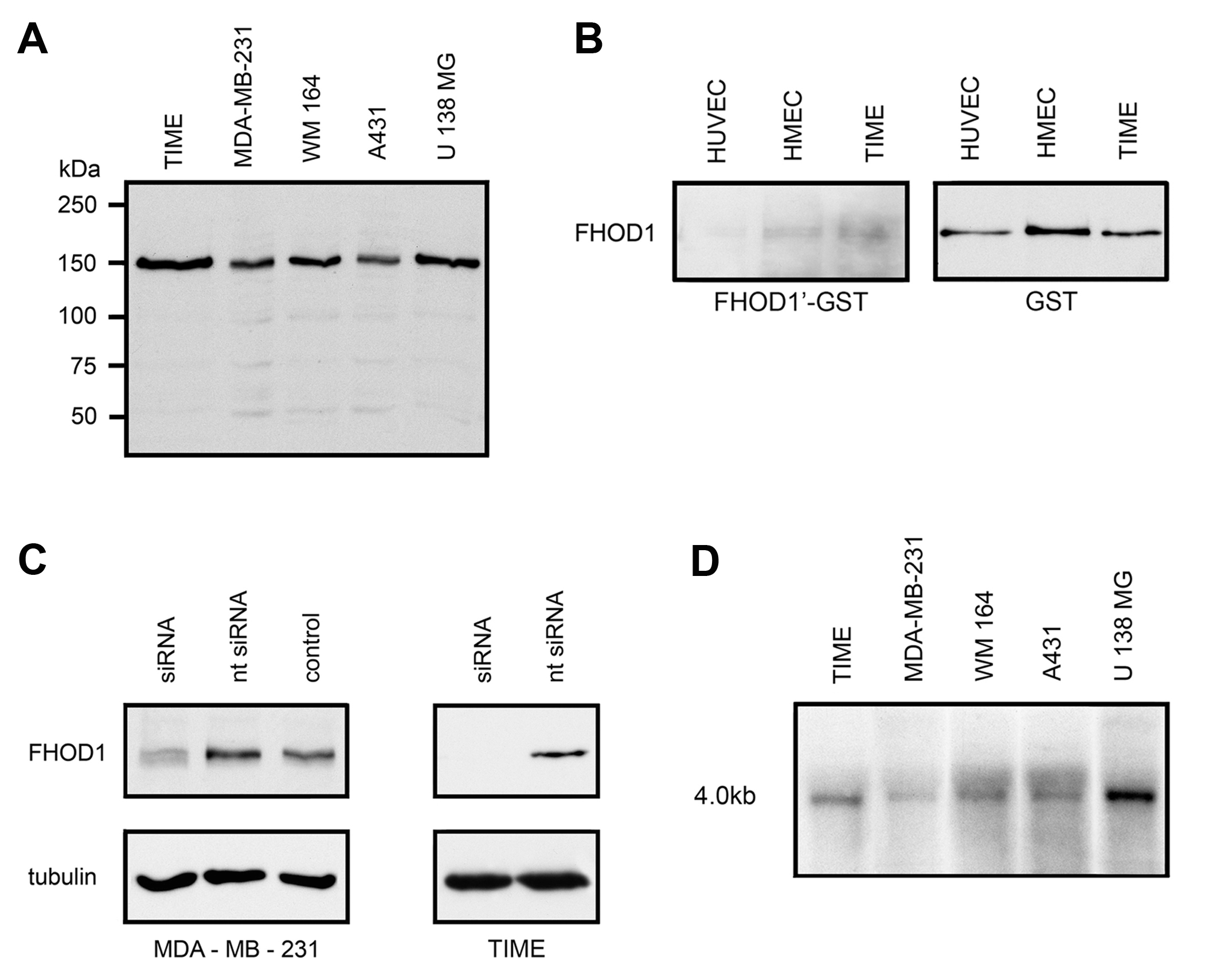

Supplement: Figure S1 — Expression of FHOD1 in cell lines and characterization of the FHOD1 antibody. A) Western blotting of lysates from four cancer cell lines and TIME endothelial cells. A single band of approximately 145 kDa is detected in all lanes. B) Preincubation of the FHOD1 antibody with a GST-FHOD1′ fusion peptide abrogates the reactivity as compared to incubation with GST alone. C) Transfection of MDA-MB-231 (left panel) or TIME (right panel) cells with FHOD1 siRNA markedly reduces the reactivity, whereas control non-targeting (nt) siRNA has no effect. D) A Northern blot analysis of the same cell lines as in a) shows a single FHOD1 4.0 kb mRNA transcript in the studied cell lines, matching the western blot result. (JPG) [file pone.0074923.s001.jpg]

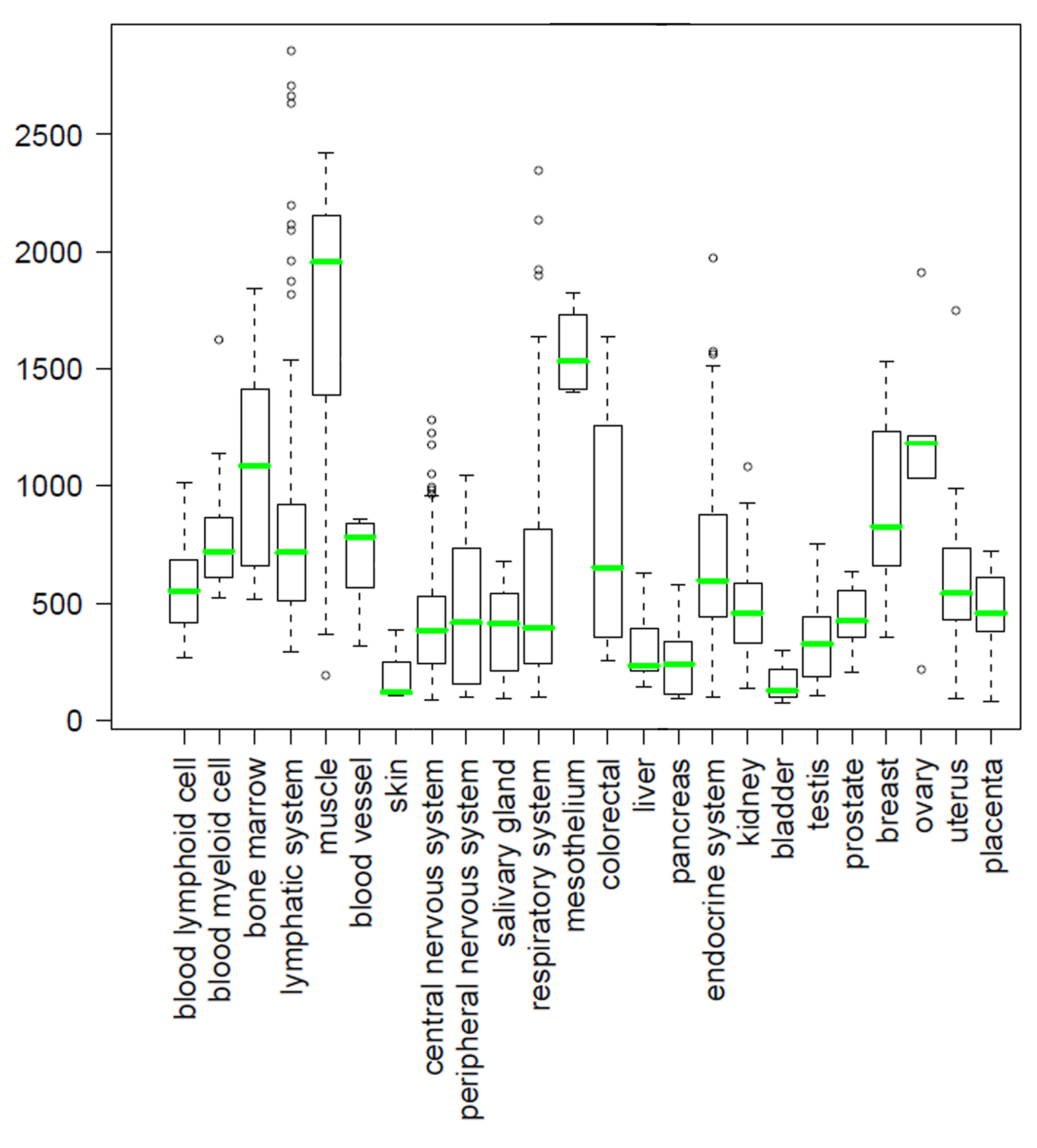

Supplement: Figure S2 — FHOD1 mRNA expression profile in normal human tissues. The expression of FHOD1 mRNA was evaluated using the GeneSapiens database information. The normalized expression values (y-axis) of FHOD1 across normal tissues (x-axis) are presented as box-plots. The box extends from the first to the third quartile of the data and the median is indicated with green. The whiskers extend to the extreme values unless there are outliers. The data observations that lie more than 1.5 * interquartile range (IQR) lower than the first quartile, or 1.5 * IQR higher than the third quartile are considered as outliers and indicated separately. Low FHOD1 expression is seen in most tissues. The highest expression levels are seen in skeletal muscle and mesothelial samples. (TIF) [file pone.0074923.s002.tif]
